# Prevalence of Computer Vision Syndrome among School-Age Children during the COVID-19 Pandemic, Saudi Arabia: A Cross-Sectional Survey

**DOI:** 10.3390/children9111718

**Published:** 2022-11-09

**Authors:** Ismail Abuallut, Reham E. Ajeebi, Alanoud Y. Bahari, Manal A. Abudeyah, Atheer A. Alyamani, Atyaf J. Zurayyir, Abdulkareem H. Alharbi, Abdullah A. Al Faqih, Abdullatif Z. Suwaydi, Maram I. Alqasemi, Bushra A. Alnami, Khaled Jamaan Al Zahrani

**Affiliations:** 1 Department of Surgery, Ophthalmology Division, Faculty of Medicine, Jazan University, Jazan 45142, Saudi Arabia; 2 Faculty of Medicine, Jazan University, Jazan 45142, Saudi Arabia; 3 Department of Preventive Medicine, Ministry of National Guard, Riyadh 22490, Saudi Arabia

**Keywords:** computer vision syndrome (CVS), COVID-19, school-age children, CVS risk factors

## Abstract

Background: Computer vision syndrome (CVS) can be described as ocular-related symptoms that result from prolonged exposure and use of computers, smartphones, tablets, and other devices with digital displays. The main objective of this study was to investigate the prevalence of CVS among school-age children, the associated signs, risk factors, and the association between the disease before and during the COVID-19 pandemic in the Jazan region of Saudi Arabia. Methods: The study employed a descriptive cross-sectional research design. The targeted population was school-going children aged 6 to 18 in the Jazan region in the Southwest of Saudi Arabia. A sample of 440 participants was selected to represent the population under study. Data were collected using self-administered questionnaires. Sociodemographic characteristics were recorded, such as age, gender, education level, parents’ education, occupation, frequency, and intensity of eye symptoms if present. Results: Most of the participants were adolescents between 16 and 18 and at a high-school education level. According to the total symptoms score, the CVS prevalence was 35.4%. Prevalence of CVS significantly affects age, gender, and school level (*p* < 0.05 for all). A similar significant association was reported between the symptoms experienced before and during COVID-19 and the CVS (*p* < 0.05). Conclusion: A total of 407 adolescents aged 16–18 responded to the questionnaire (response rate of 92.5%; 407 out of 440). The study estimated the prevalence of CVS among school-going children in Jazan to be low. The main signs associated with CVS included headache, tearing, itchiness, blurred vision, eye redness, eye pain, and dryness. The attitude of children toward their health condition during the COVID-19 pandemic and the prevalence of CVS have a significant relationship.

## 1. Introduction

Computer vision syndrome (CVS) has become a significant public health issue across all age groups due to the increased use of electronic devices, such as computers, laptops, smartphones, tablets, and e-readers [1,2].

The American Optometric Association defines CVS as a group of complications correlated to vision and eye that result from prolonged digital screen view [1,3]. This syndrome is associated with various symptoms, such as “eye strain, blurred vision, dry eyes, headache, and neck and shoulder pain”. CVS can also lead to eye irritation, redness, or burning [4]. Using a computer or any digital device for more than 3 h a day may lead to an experience of CVS. Furthermore, approximately 60 million individuals are diagnosed with CVS globally, where a million new CVS cases transpire annually [5].

Children spend more time with modern and highly advanced media devices, such as computers, tablets, and smartphones than traditional media, such as printed publications, television, and radio. Contemporary studies revealed that more than two-thirds of children surpass the recommended screen time (2 h/day) issued by the American Academy of Pediatrics [6,7].

Children who spend extended periods on computers and electronic devices may have the same problems as adults; however, how they use them may make them more susceptible [7]. According to a study conducted in Iran, eye pain, redness, and other CVS symptoms are the most common problem associated with prolonged use of electronic devices [8]. Another study conducted in Korea showed that increased exposure duration to screens poses a higher risk of experiencing eye symptoms [9].

The predominance of CVS and its allied risk factors in Saudi Arabia were detected by several studies performed in Jeddah, Riyadh, Najran, and Qassim, demonstrating relatively high prevalence (95%) among different population groups, and among undergraduate medical students in Jeddah [10]. Furthermore, 68% of the students in health sciences at King Saud Bin Abdul-Aziz University experienced headaches as a significant symptom of CVS [11]. In Riyadh, approximately 82.2% of university students complained of neck and shoulder pain [12]. In Najran, 62% of computer users reported blurred vision [13]. At Qassim University, 72% of college students presented with acute CVS symptoms [14].

In response to the coronavirus disease 2019 (COVID-19) pandemic, quarantine measures have been implemented worldwide. Children spend more time than ever on electronic devices when working, schooling, or just trying to pass time [15]. This rapid increase in screen time can also significantly increase the number of children suffering from CVS. Unfortunately, only limited studies have evaluated the predominance of CVS among school-age children globally, and no study has been conducted in Saudi Arabia [16,17,18]. Hence, the main aim of this study was to estimate the prevalence of CVS and its allied risk factors among school-age children during the COVID-19 pandemic.

The main objective of this study was to investigate the prevalence of CVS among school-age children, the associated signs and risk factors, and the association between the disease before and during the COVID-19 pandemic in the Jazan region of Saudi Arabia.

## 2. Materials and Methods

### 2.1. Study Design and Setting

A descriptive cross-sectional study was used to establish the association among the symptoms, risk factors, and the attitude of participants to examine the prevalence of CVS among school-going children. This study was conducted in Jazan, Saudi Arabia, the second smallest region in the country. It covers an area of 11,671 km and has a population of 1,567,547.

### 2.2. Sampling Procedure

This study targeted school children with an age ranging from 6 to 18 years old. According to the 2018 general authority for vital statistics, the total population between the ages of 6 and 19 was 416,631. All students who registered for the academic year 2020/2021 were targeted for this survey. The exclusion criteria were ophthalmological disorders, such as refractive errors and strabismus. In addition, since the general education system in Saudi Arabia generally includes healthy children, children with special needs are usually enrolled in special schools that cater to their needs. We included school-age children in the Jazan region in the selected age group between 6 and 18 years old who registered for the academic year 2020/2021.

A sample of 440 participants was estimated for this study; the sample size was calculated using the sample size formula [19], *n* = (z)^2^ *p* (1—*p*)/d^2^. The following parameters were utilized to calculate the sample size: *p* = prevalence of knowledge of 50%, Z = 95% confidence interval, d = error of no more than 5%, and a 15% nonresponse rate (the nature of online surveys requires a large sample size as the nonresponse rate is always high. The sampling plan utilized the administrative distribution of schools in the Jazan region (Jazan Administration for Education and Sabya Department of Education). We employed convenient sampling to choose 12 schools from Jazan and Sabya Education offices. From each educational level, one male school and one female school were selected. After securing the school administration’s permission, a group of students in each school was selected conveniently, and the survey was sent to their school emails.

### 2.3. Data Collection and Study Tools

Questionnaires were designed and sent to experts for content validity before administering to the students. The questionnaires used were standardized to provide consistency, reliability, and validity in quantitative analysis. The data were collected via an online self-administered questionnaire sent to the sample of the participants selected through their school emails provided by the ministry of education to access their educational school platform. Parents of children in primary schools were instructed to fill the questionnaire sent to their children email. Sociodemographic characteristics, such as age, gender, education level, parents’ education, and occupation, were assessed first. The primary endpoint of the questionnaire was to determine the prevalence of CVS using the frequency and intensity of the symptoms experienced by the participants according to the Computer Vision Syndrome Questionnaire (CVS-Q) [20]. The frequency was evaluated as follows for each of the 16 components of the questionnaire: never, which means no symptom at all and is given a score of 0; occasionally, which means sporadic or once a week and is given a score of 1; often, which means at least twice weekly and is given a score of 2. The intensity was evaluated similarly but had only two categories: moderate is given a score of 1; intense is given a score of 2. Then, for each symptom, the frequency score was multiplied by the intensity score, and the result was adjusted as follows: 0 = 0; 1 or 2 = 1; 4 = 2. The recorded result for each of the 16 symptoms was added to give a total score. A total score >6 was considered a CVS case.

### 2.4. Data Analysis

The standard computer program IBM SPSS Statistics for Windows, version 20.0 (IBM Corp., Armonk, NY, USA) was used to enter, organize, tabulate, and analyze data. Social status and demographic data were tabulated and articulated as the percentage and frequency of the total participants. Pie charts and bar graphs were used to illustrate the frequency distribution of the different variables. The chi-square test was used to test the significance of the association of the stated variables. The analysis covered the significant association between the prevalence of CVS and demographic characteristics, the relationship between CVS symptoms before and during the COVID-19 pandemic, and the opinion of the students toward their health status performance during the COVID-19 pandemic period. Multivariate logistic regression analysis was used to assess the participants’ independent predictors (risk factors) of CVS. A *p*-value less than 0.05 was considered statistically significant.

### 2.5. Ethics and Confidentiality

Ethical approval was sought from MOH IRB. The questionnaires were reviewed and approved by experts before they were administered. Consent was sought from the parents and schools before involving the children in the survey. The data were used under strict confidentiality and were only used for research purposes.

## 3. Results

From the target sample, 407 participants responded to the questionnaire (response rate of 92.5%; 407 out of 440). According to Table 1, the female participants were the majority and accounted for 51.4%, while the males constituted 48.6%. The ages of 16 to 18, 13 to 15, and 6 to 12 accounted for 47.4%, 27.5%, and 25% of the total, respectively. Concerning their education level, 45.7% were in high school, 29.5% were in middle school, and 24.8% were in primary school. Most of the children were from the city side, accounting for 62.2%, whereas those from the village were 37.8%. According to the table, most parents had acquired a bachelor’s degree, 57.25% of mothers were housewives, 34.6% were employed in the private sector, and the remainder were either retired or unemployed. Furthermore, 52.6% of the fathers were in the government sector, 25.8% were retired, 13.3% were in the private sector, and the remainder were unemployed.

Table 2 presents the prevalence of CVS according to demographic and other characteristics. The overall prevalence of CVS was 35.4% (CVS scores ≥ 6), while 64.6% of the participants passed the threshold and were classified without computer vision syndrome. The table revealed a significant association between CVS and age, gender, and school level (*p* < 0.05 for all). The prevalence of CVS reached 54.1% for children with an age range from 16 to 18 years. Females had a higher prevalence of CVS at 40.2%, compared with males at 30.3%. Participants in the high schools reported a high prevalence among the students, 43.0%. There was no significant association between the prevalence of CVS and participants who had chronic diseases (*p* = 0.962). There was a significant association between CVS prevalence and patients who reported symptoms before the COVID-19 pandemic (*p* < 0.05).

**Figure 1:**
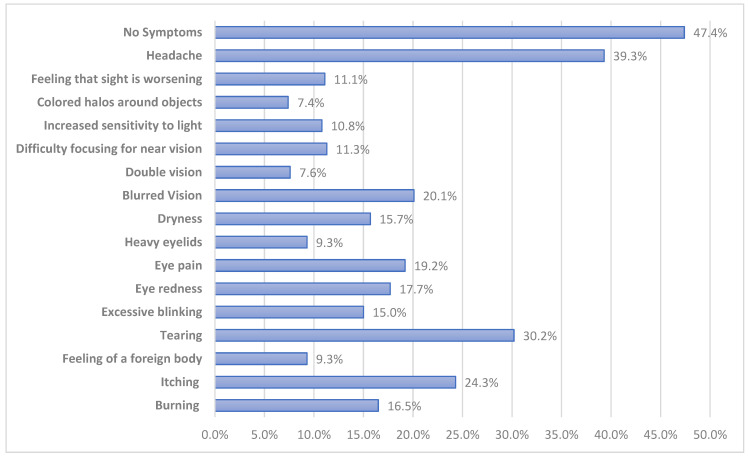
Frequency of eye symptoms reported during COVID-19.

Figure 1 illustrates eye symptoms during COVID-19 as reported by the study participants. According to the figure, headache was the most selected symptom at 39.3%, followed by tearing at 30.2%, itching at 24.3%, blurred vision at 20.1%, eye pain at 19.2%, eye redness at 17.7%, burning at 16.5%, excessive blinking at 15.0%, and dryness at 15.7%. Other minor selected symptoms were double vision and colored halos around objects.

**Figure 2:**
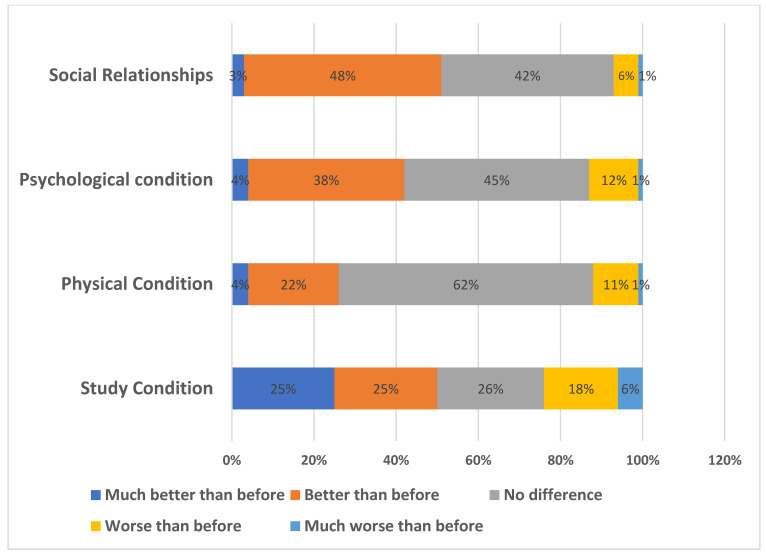
Pupils’ responses regarding a change in some conditions after COVID-19.

Regarding their study condition, 26% of participants reported no difference or change, 50% said it was much better than before, and only 6% said it was much worse. Regarding their physical condition, 62% reported no change, whereas only 1% said it was much worse than before. A total of 45% of participants reported no difference in their psychological condition, while 48% reported a better social health condition than before (Figure 2).

Table 3 demonstrates the independent risk factors of CVS among the study participants. Female gender was significantly associated with an increased risk of CVS as compared with males (OR = 1.5, 95% CI = 1.0–2.3, *p* = 0.038), and students in the higher age groups were significantly associated with a high prevalence of CVS as compared with participants in the age group of 13 to 15 years (OR higher than 1 for all). Students at intermediate and secondary schools were also at high risk of CVS (OR = 2.0, 95% CI = 1.2–3.3, *p* = 0.006 and OR = 1.7, 95% CI = 1.0–2.8, *p* = 0.042, respectively). Lastly, symptoms experienced before COVID (OR = 3.6, 95% CI = 2.4–5.5, *p* < 0.001) and increased severity of symptoms with COVID (OR = 7.8, 95% CI = 4.9–12.4, *p* < 0.001) were also independent predictors (risk factors) of CVS among the studied participants.

## 4. Discussion

This study aimed to investigate the prevalence of CVS among school-going children in the Jazan region of Saudi Arabia. The prevalence was examined before and after the COVID-19 pandemic. Furthermore, the associated risk factors, signs, and children’s attitudes toward time spent on computers during the COVID-19 pandemic were examined. An associated risk factor is long smartphone exposure, especially in adolescents with prior ocular symptoms [9]. Similar results by [21] suggested that the risk factors included higher daily computer usage and longer duration of exposure, as well as pre-existing eye disease, among Sri Lanka computer workers. Another study [22] also reported that the risk factors for CVS among computer workers in Debre Tabor town, Northwest Ethiopia, included daily computer usage and pre-existing eye disease. Furthermore, the authors of [23] studied undergraduate medical students at Bahria University Medical Dental, Karachi, and found similar associated risk factors. Another study [24] suggested similar risk factors to the current research. However, only one study [11] found that a long duration of exposure was not an associated risk factor among students at King Saud Bin Abdulaziz University of Health sciences in Jeddah. Therefore, considering the extant literature and the current study, it can be concluded that the main risk factors for CVS are long computer usage and pre-existing eye disease.

Regarding the signs and symptoms of CVS, headaches were the most selected symptoms by the participants, closely followed by tears. Other significant cases of itching, blurred vision, redness, eye pain, and excessive blinking were reported. A study by [25] on computer vision problems among university students suggested that headache was the main symptom reported because of the extensive use of computers for long hours, which resulted in eye fatigue. The authors of [26] also reported blurred vision and eye redness among medicine students. Other studies [27,28] reported eye dryness as one of the symptoms of CVS. Therefore, on the basis of evidence from the literature and the current study, it can be concluded that the probability of occurrence of eye symptoms in children with CVS is high.

A cross-section analysis between the prevalence of CVS and demographic characteristics showed a significant association with age, gender, and education level. Most students who suffered from CVS were 16 to 17 years old and were in high school. The number of female participants was also higher than that of their male counterparts. The study points to female children being at a greater risk of developing CVS. Similar results were reported in [11], where female students reported higher scores in eye symptoms than male students. In an age study [29] on the prevalence of CVS among school-going children in China, a high CVS score was associated with older children. Another study conducted in India [30] found that an age of more than 14 years is considered a risk factor due to increased screentime in older children [31] Therefore, on the basis of evidence from the existing literature and the current study, it can be concluded that age, gender, and education level play a major role in the prevalence of CVS among children.

The current study established a significant association between the prevalence of CVS and symptoms experienced before and during the COVID-19 pandemic. In addition to headache as the main sign reported, tearing and itchiness of the eyes were among the most commonly reported symptoms. In a previous study [29], the most typical symptoms reported were itchiness and dryness of the eye. The reported main risk factors were a pre-existing ocular disease, myopia, excessive screen time, and reduced outdoor activities. Similar reported results were established in studies by [28,29].

Although the prevalence of CVS and the attitude of children toward their health status are significantly associated, most of the children reported no difference in terms of their physical, psychological, social, and health status in their study. Most children do not have such a problem because of the lack of the knowledge regarding their physical, psychological, social, and health status [32]. A significant number of children are still naïve and might not be aware of their health status. However, it is difficult to assess the impact on physical, psychological, and social status due to the scarcity of studies conducted before COVID-19 for comparison with our results.

The main limitation of this study is that it was a cross-section study that only covered children of two institutions in the Jazan area. Hence, the findings are only limited to the selected sample, thereby limiting the generalization of the conclusion to the whole Jazan area. Furthermore, self-reporting was another limitation of the study; future studies should incorporate ophthalmic examination and parent interviews. This study did not examine the direct link between prolonged computer use and CVS. Therefore, to establish causation, more studies must be conducted to determine the effect of spending more time on digital computer screens and the prevalence of CVS.

## 5. Conclusions

In conclusion, among the 440 children, 407 answered the questionnaires, and 47.4% of the children were adolescents between 16 and 18 years at a high-school education level. The study estimated the prevalence of CVS among school-going children in Jazan to be low, accounting for 35.4%. A significant association between prevalence and demographic factors, such as age, gender, and education level, was established. A significant association between the prevalence of CVS and symptoms before and during the COVID-19 pandemic was also established. The main signs associated with CVS included headache, tearing, itchiness, blurred vision, eye redness, eye pain, and dryness.

## Figures and Tables

**Table 1:** Demographic characteristics of the study participants.

Variables		N	%
Gender	Girls	209	51.4%
	Boys	198	48.6%
Age	6 to 12 years old	102	25%
	13 to 15 years	112	27.5%
	16 to 18 years old	193	47.4%
Education level	High school	186	45.7%
	Middle school	120	29.5%
	Primary stage	101	24.8%
Education department	Jazan Education Department	128	31.4%
	Sabya Education Department	279	68.6%
City/village	City	253	62.2%
	Village	154	37.8%
Mother’s education level	Bachelor’s/associate degree	210	51.6%
	Primary	69	17.0%
	Secondary	78	19.2%
	Uneducated	50	12.3%
Father’s education level	Bachelor’s/associate degree	206	50.6%
	Primary	53	13.0%
	Secondary	114	28.0%
	Uneducated	34	8.4%
Mother’s job	An employee in the government sector	141	34.6%
	An employee in the private sector	13	3.2%
	Housewife	233	57.2%
	Retired	20	4.9%
Father’s job	An employee in the government sector	214	52.6%
	An employee in the private sector	54	13.3%
	Retired	105	25.8%
	Unemployed	34	8.4%
Chronic disease suffered	Asthma	17	4%
	Diabetes	17	4%
	Hereditary blood disease	6	2%
	Other	16	4%
	Do not have any chronic disease	355	87%
	Asthma	17	4%
Total		407	100

**Table 2:** Prevalence of CVS according to some selected characteristics.

Variables		Computer Vision Syndrome		*p*-Value
		Presence of CVS	Absence of CVS	
Age	13 to 15 years	30.4%	69.6%	0.020
	16 to 18 years	54.1%	45.9%	
	6 to 12 years	32.0%	68.0%	
Gender	Female	40.2%	59.8%	0.037
	Male	30.3%	69.7%	
School level	High school	43.0%	57.0%	0.011
	Middle School	27.5%	72.5%	
	Primary school	30.7%	69.3%	
City/village	City	35.6%	64.4%	0.917
	Village	35.1%	64.9%	
Mother’s job	Government sector	37.6%	62.4%	0.424
	Private sector	15.4%	84.6%	
	Housewife	34.8%	65.2%	
	Retired	40.0%	60.0%	
Father’s job	Government sector	37.9%	62.1%	0.107
	Private sector	35.2%	64.8%	
	Retired	26.7%	73.3%	
	Unemployed	47.1%	52.9%	
Suffered from chronic disease	No	35.3%	64.7%	0.962
	Yes	35.7%	64.3%	
Experienced symptoms before COVID	No	23.9%	76.1%	0.000
	Yes	53.1%	46.9%	
The severity of symptoms increased with COVID	No	18.8%	81.2%	0.000
	Yes	64.8%	35.2%	
Overall prevalence		35.4%	64.6%	

**Table 3:** Logistic regression analysis of risk factors of CVS among the pupils.

Factors	Logistic Regression Model			
	*p*-Value	OR	95% C.I. OR	
			Lower	Upper
Gender				
Male	Ref	1		
Female	0.038	1.5	1.0	2.3
Age groups				
13 to 15 years	Ref	1		
16 to 17 years	0.003	2.7	1.4	5.2
6 to 12 years	0.009	2.3	1.2	4.2
School Level				
Primary	Ref	1		
Intermediate	0.006	2.0	1.2	3.3
High School	0.042	1.7	1.0	2.8
Experienced symptoms before COVID				
No	Ref	1		
Yes	<0.001	3.6	2.4	5.5
The severity of symptoms increased with COVID				
No	Ref	1		
Yes	<0.001	7.8	4.9	12.4

## Data Availability

The data that support the findings of this study are available upon request from the corresponding author.
